# Supplementary material for: Early onset of aridity in the past millennium: Insights from vegetation dynamics and climate change in the alpine, cold-desert region of Trans Himalaya, India
Source: PLoS One. 2024 Jan 10;19(1):e0295785. doi: 10.1371/journal.pone.0295785 (PMC10781162; doi:10.1371/journal.pone.0295785)
Supplement: S1 Table — (PDF) [file pone.0295785.s004.pdf]

S.No.	Sample No.	Depth (cm)	Weight of sample (w)	Magnetic Susceptibilty (χlf)
1.	HOA−1	85−90	4.509	3.386
2.	HOA−2	80−85	4.465	4.613
3.	HOA−3	75−80	4.885	4.360
4.	HOA−4	70−75	4.416	4.438
5.	HOA−5	65−70	5.185	4.108
6.	HOA−6	60−65	4.438	7.503
7.	HOA−7	55−60	5.018	6.237
8.	HOA−8	50−55	4.709	7.921
9.	HOA−9	45−50	4.448	2.922
10.	HOA−10	40−45	5.072	6.230
11.	HOA−11	35−40	4.851	2.391
12.	HOA−12	30−35	6.277	3.441
13.	HOA−13	25−30	4.523	4.302
14.	HOA−14	20−25	4.079	5.393
15.	HOA−15	15−20	3.823	6.263
16.	HOA−16	10−15	3.967	8.721
17.	HOA−17	5−10	4.582	5.368
18.	HOA−18	0−5	3.182	8.170
