# Supplementary figures and images for: Early onset of aridity in the past millennium: Insights from vegetation dynamics and climate change in the alpine, cold-desert region of Trans Himalaya, India
Source: PLoS One. 2024 Jan 10;19(1):e0295785. doi: 10.1371/journal.pone.0295785 (PMC10781162; doi:10.1371/journal.pone.0295785)

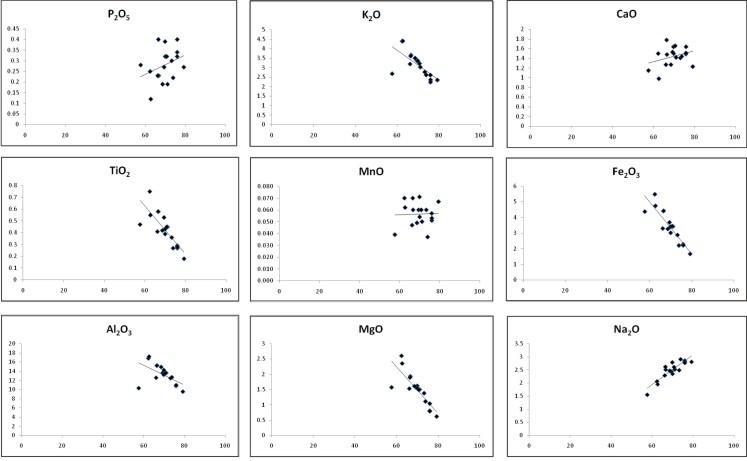

Supplement: S1 Fig — (JPG) [file pone.0295785.s005.jpg]
