# Supplementary material for: Early onset of aridity in the past millennium: Insights from vegetation dynamics and climate change in the alpine, cold-desert region of Trans Himalaya, India
Source: PLoS One. 2024 Jan 10;19(1):e0295785. doi: 10.1371/journal.pone.0295785 (PMC10781162; doi:10.1371/journal.pone.0295785)
Supplement: Attachment — Submitted filename: To the editor.pdf [file pone.0295785.s007.pdf]

To the editor:

The study provides a valuable scientific contribution by documenting a retrospectively prolonged duration of LIA in the Trans Himalaya region. The authors have efficiently compared the timeframes of the four major climatic events (DACP, MCA, LIA and CWP) inferred from their study area with those reported from other parts of Himalaya. The climatic events have been suggested to be asynchronous in nature.

However, I was just wondering if there is any correlation between the transgression of aridity over a region and its latitude or altitude. To make it simple, if the Trans Himalaya received aridity prior to the Greater Himalaya followed by the Lesser Himalaya and Siwalik. It need not be a component of the current study; nevertheless, the editor may suggest it to the authors for future consideration.

I have suggested the authors to incorporate some minor edits in the manuscript.
