# Supplementary material for: Early onset of aridity in the past millennium: Insights from vegetation dynamics and climate change in the alpine, cold-desert region of Trans Himalaya, India
Source: PLoS One. 2024 Jan 10;19(1):e0295785. doi: 10.1371/journal.pone.0295785 (PMC10781162; doi:10.1371/journal.pone.0295785)
Supplement: Attachment — Submitted filename: Response to Reviewers.doc [file pone.0295785.s008.doc]

**Response to Reviewers**

**Academic editor**

1. Please ensure that your manuscript meets PLOS ONE's style requirements, including those for file naming.

**Response: We understand that the manuscript is according to PLOS ONE's style requirements.**

2. In your Methods section, please provide additional information regarding the permits you obtained for the work. Please ensure you have included the full name of the authority that approved the field site access and, if no permits were required, a brief statement explaining why.

**Response: No permits were required regarding field access. The work was one of the components of the in-house projects of the Institute, which are already approved. We have acknowledged the Director (competent authority) of the Institute for the facilities provided and the permission to publish.**

3. Thank you for stating the following in the Acknowledgments Section of your manuscript:

"RBM is grateful to the ………….(DST/CCP/PR/07/2011/G) under which field work and sampling were done."

We note that you have provided additional information within the Acknowledgements Section that is not currently declared in your Funding Statement. Please note that funding information should not appear in the Acknowledgments section or other areas of your manuscript. We will only publish funding information present in the Funding Statement section of the online submission form.

**Response: Have modified the Acknowledgements Section, as suggested.**

Please remove any funding-related text from the manuscript and let us know how you would like to update your Funding Statement. Currently, your Funding Statement reads as follows:

"RBM is grateful to the ……………….under the Climate Change Programme (DST/CCP/PR/07/2011/G) "

Please include your amended statements within your cover letter; we will change the online submission form on your behalf.

**Response: We have removed the funding-related text from the manuscript and included our statement in the cover letter.**

4. Thank you for stating the following financial disclosure:

"RBM is grateful to the ………………under the Climate Change Programme (DST/CCP/PR/07/2011/G) "

Please state what role the funders took in the study.  If the funders had no role, please state: "The funders had no role in study design, data collection and analysis, decision to publish, or preparation of the manuscript."

**Response: We have stated the above in the cover letter.**

5. We note that Figure 1 in your submission contain map images which may be copyrighted………

**Response: May we emphatically state that Figure 1 is NOT copyrighted. Figure 1a & b were made using Corel Draw, while Figure 1c was made using ArcGIS 10.3 (also stated in the figure caption).**

6. Please review your reference list…………

**Response: No changes made in the reference list and also those cited in the manuscript.**

**Reviewer #1**

We are grateful to the esteemed reviewer for meticulously going through the manuscript and for the very useful suggestions. We are pleased to see that he/she has found the paper ‘quite well written’ and for the encouraging comments. We have incorporated most of the suggestions; at few points we have differed and have given plausible explanations for the same.

The point wise response to each query is given below:

1. The title has an emphasis on early onset of aridity in the Trans Himalayan region however, this is not very explicit in the text- results as well as discussion. The authors need to highlight the points based on which they have arrived to this finding. The discussion on early inception of aridity is very subtle and not very convincing. Changes need to be made in the text/ title accordingly.

**Response: Well, we would like to slightly differ here. An early onset of aridity in the Trans Himalayan region, at the beginning of the last millennium, is the novel finding of the present work. At 950 yr BP, there is a prominent decline in the AP/NAP ratio (from 82% to 65%), which clearly demarcates the two palynozones. This sharp fall in the frequency of arboreal pollen is the manifestation of the onset of cold-arid conditions, which are rather a little too early in this Trans Himalayan region, as compared to the other well-known records. We feel that we have provided robust data to back our inferences. Please see sections under Results and interpretations; sub-head Palynological analysis of the sub-surface sediments; Pollen zone-III; and also Discussion: 950 yr BP to the Present, 1^st^ paragraph). Hope that we have provided satisfactory replies to the above queries and the esteemed reviewer would approve the same.**

**Please also see our response to your point’s no. 2 & 3 below.**

2. Do the authors prefer to suggest early initiation of LIA and then put the terminal phase of MCA? The authors need to provide strong evidence as to why cannot it be interpreted as a period of aridity within MCA? The MCA is known for fluctuating precipitation and temperature conditions. Event based deposition or constant sedimentation rate?

**Response: Yes, we are suggesting an early inception of LIA in the Trans-Himalayan region. You are right that ‘The MCA is known for fluctuating precipitation and temperature conditions.’ Nevertheless, this phase cannot be interpreted as a ‘period of aridity within MCA’, but rather as a different climatic phase, because the AP/NAP ratio registers the sharpest fall here (from 82% to 65%). Other than the AP/NAP ratio, the climatic distinction between this colder phase and the earlier warmer one is further supported by the proliferation of arid-loving *Artemisia* and lesser frequency of moist-loving ferns. Besides, the magnetic susceptibility and geochemical data also indicate a turnover from warmer to colder conditions. We have clearly presented the data for the same (please see Discussion: 950 yr BP to the Present, 1^st^ paragraph).**

3. The change in the climatic conditions from warm-moist to cold-arid has been made largely based on the percentage of broad-leaved taxa rather than the AP/NAP ratios. It may be helpful to include that detail in summary figure rather than AP/NAP ratio as these remain almost the same throughout the sediment profile.

**Response: We beg to differ somewhat! ‘The change in the climatic conditions from warm-moist to cold-arid’ has been done both on the basis of AP/NAP ratios, aided by the percentage of broad-leaved taxa. Your observation that the ‘AP/NAP ratio remain almost the same throughout the sediment profile,’ is for the lower half of the profile only (in the earlier two palynozones), where the AP/NAP ratio is almost similar (AP: 81% and 82%, respectively). However, these two zones can be distinguished by the change in the frequencies of broad-leaved elements (6% and 9%, respectively). Even subtle changes in the frequencies of the temperate broad-leaved taxa, which are thermophilic in nature, are considered good indicators of climatic changes in the Higher Himalayan region. Moreover, there is a sharp fall in the AP/NAP ratio between the second and third palynozones (AP: 82% and 65%, respectively). We have not put the changing frequencies of broad-leaved taxa in the summary diagram, but have rather discussed the above data comprehensively in the section under ‘Results and interpretations’; sub-head ‘Palynological analysis of the sub-surface sediments.’**

4. Chronology is discussed in BP as well as AD and also centuries which makes it difficult and confusing for the reader to follow… uniformity is easy for correlation.

**Response: We have given our ages in BP in the Chronology head. Most of the chronology has been discussed in BP, as cited in the respective works. Only in certain cases we have additionally given ages in AD in brackets, as these have appeared in the original publication, for correlation and clarity. Nonetheless, there were few minor discrepancies, which we have addressed.**

Some specific points-

Lines 15-16- this is not seen in the results. For more than half of the trench depth, the values of AP/NAP remain almost same.

**Response: We have answered the same in point no. 3 above. We had missed to mention about the importance of broad-leaved taxa here, so we added a few words regarding the same – ‘complemented by the frequencies of the broad-leaved taxa.’**

Line 17- you mean lower values not lesser

**Response: Yes.**

Lines 22-24- “This cold-arid phase was, nevertheless, punctuated by a warm-moist period during 790 to 680 yr BP (AD 1160−1270), which marks the termination of the MCA. The full extent of the Little Ice Age (LIA) is well marked in the area.” Does this mean that the authors suggest beginning of LIA prior to termination of MCA?

**Response: Yes, as pointed out earlier, the main premise of the paper is the early initiation of a cold-arid phase in the region, which is manifested by the longer duration of the LIA and well recorded by the different proxies used in the present study. Whereas the warmer phase, represented by the MCA, is not that prominent. Nonetheless, the palynological data has recorded a short warming episode between 790 and 680 yr BP, which we postulate as the culmination phase of the MCA, as it matches well with the ‘termination of the MCA’ as correlated with the global and Indian records.**

Line 28- evidence

**Response: Thanks for correcting.**

Line 29- late Holocene or last 1500 years?

**Response: Ok, the last 1580 years!**

Lines 30-31 delete.

**Response: Ok, fine!**

Lines 56-73- proxies are known… not needed in introduction… can go to methods section if absolutely necessary.

**Response: Beg your pardon, but we feel that these lines are better suited in the Introduction, where we introduce the tools (proxies) used in the present study. It is true that the different ‘proxies are known’; however, just a few lines about these may please be allowed.**

Lines 86-88- do geographical boundaries matter?

**Response: Well, ‘geographical boundaries do not really matter’, but often the reviewers/editors ask for specifics about the area of study, specially for the benefit of non Indian readers. So we think that a couple of lines are OK.**

Lines 109-110- refer Fig. 2a

**Response: Done, as suggested.**

Line 173- delete ‘.’ Before d.

**Response: Yes.**

Lines 220-222- not reflected in the results and figure.

**Response: I think we have addressed similar queries earlier – please refer to point no. 3 and your query regarding Lines 15-16. Besides, Reviewer #2 has suggested rephrasing the same sentences, which we have complied with.**

Lines 280-292- Why is Pinus pollen the most abundant in surface as well as subsurface samples? Has this anything to do with the overall climatic conditions of the region?

**Response: Thanks for pointing this out; we had missed to write reasons for the abundance of *Pinus* pollen. We have extensively worked in the Himalayan region and everywhere we have encountered the predominance of *Pinus* pollen in the palynological assemblages (References already given: 26, 27). We have added the following lines in the text:**

***Pinus* spp. are prolific pollen producers and owing to their excellent buoyancy, are efficiently transported by winds to long distances. They also have good preservation potential in the sediments, which enables them to be bountifully recorded in the palynological assemblages. It has been observed that even if *Pinus* is not growing in the immediate vicinity of the sampling locations, its pollen can be predominant to such an extent that it completely overwhelms the other pollen taxa in the palynological records.**

Lines 294-300- The grain size is already mentioned in the description of three palynozones.

Why repeat? Lot of it is methodology. This part along with the ternary diagram can be added to the methodology section.

**Response: It is not really a repetition. In the Methodology section, the chemical processing of the samples and instrument involved were briefly mentioned. Whereas here, a few lines about the utility and need for grain size analysis and the results obtained thereby, have been given.**

Lines 327-329--- this is similar to present day-

**Response: Perhaps you mean that the AP/NAP ratio is similar to the present day (CWP)? If that is so, then it is just a coincidence. We have faithfully presented our data.**

Lines 452-498--- what do the authors want to highlight here? Are they discussing the time duration of the late Holocene climatic events?... there has never been any consensus as there are local factors governing. Such a discussion can be avoided.

**Response: Yes, your observations are correct that as far as the late Holocene climatic events are concerned “there has never been any consensus as there are local factors governing.” However, do allow us to say here that there was lack of global contextualisation of the Indian records, as the earlier works were not of good resolution. Nonetheless, during the last two decades, or so, good high-resolution multi-proxy, Holocene climatic records have been generated from India. Therefore, our attempt is to highlight the Indian data and correlate these with the well-known global records, so as to have global connotation of the Indian works. Hope you would agree to our point of view.**

Line 505- conclusion 3- reduced values? As compared to what?

**Response: Yes, we agree that it is confusing, so we have deleted the said part and corrected the sentence.**

Line 514- Add ‘( ‘ between BP and AD.

**Response: Right, thank you.**

Line 518- Delete additional full stop.

**Response: Yes.**

Figure 10- shows overlap of chronology (present study) between DACP – MCA and MCA-LIA. Please check.

**Response: Yes, the ‘overlap’ is due to the asynchronicity of the Late Holocene climatic events: DACP. MCA, LIA and CWP regarding their inception and duration, which the esteemed reviewer was referring to earlier. However, we wanted to show the Late Holocene climatic events from India in a single figure, along with the present work and the important Global records.**

**-----------------------------------------------------**

**Reviewer #2**

We are indeed thankful to the learned reviewer for sparing his/her valuable time and for rectifying our errors. We are pleased to note that he/she has appreciated our efforts, and supported our inferences, especially the ‘backward extended period of LIA in the Trans Himalaya region.’

We have addressed all the suggestions/corrections, which are as under:

Line 42: multi-proxy.

**Response: Rectified**

Line 49: Suggested paraphrase: The fluctuating intensities of the climate systems (ISM and WDs) have had variedly impacted the Himalayan and South Asian regions.

**Response: Done as suggested, thank you!**

Line 51: Suggested paraphrase: the fusion of Higher or Greater Himalaya with Pir Panjal Range in north-western Himalaya forms a.....

**Response: Rectified accordingly.**

Line 79: Use either sub-continent or subcontinent throughout the text.

**Response: Ok**

Line 97: The initials of the abbreviation (SRTM) should be in upper case.
**Response: Yes, thank you!**

Line 120: Suggested paraphrase: Sporadic, low intensity ISM showers occur from July to September, whereas most of the precipitation falls as snowfall from November to March due to the WDs.

**Response: Ok, fine**

Line 135: Use either win-ward or windward throughout the text.

**Response: Yes**

Line 188: Use either gm or g throughout the text, as per the journal's format.
**Response: OK**

Line 218: Suggested paraphrase: From bottom to top, three pollen zones (Pollen zone-I, Pollen zone-II and Pollen zone-III) have been demarcated in the sequence based on the increase or decrease in the percentages of arboreal and non-arboreal pollen (here represented as ratio of arboreal/non-arboreal pollen).

**Response: Rephrased as suggested, thanks for the same.**

Line 246: Replace comma with the full stop and make the following text a separate sentence (However, Juniperus is present only in one sample.)

**Response: Done accordingly**

Line 263: Replace “usual” with “in the previous zone”

**Response: Ok**

Line 286: ….in all the “surface as well as sub-surface” samples. Strike out the remaining text.

**Response: Rectified as suggested**

Line 286: Suggested paraphrase: Moreover, the high proportion of Pinus pollen, along with significant similarity in the palynological assemblages from both the realms enabled......

**Response: Rephrased as suggested**

Line 321: The observed variations in the values of weathering indices throughout the trench reflect the changes in weathering intensity.

**Response: Rectified accordingly**

Line 336: ….the two can “be” observed.

**Response: Yes**

Line 372: AD is written before the values. Maintain the format throughout the text.

**Response: Yes**

Line 375: Suggested paraphrase: The maximum expansion of thermophilous broad-leaved forests from the Chandra Valley, around Kunzum La, during 1609−1303 yr BP has been correlated with the MCA.

**Response: Rephrased as suggested**

Line 405: Close the parenthesis after Present.

**Response: Yes**

Line 475: It should be 1300.

**Response: Yes, thank you**


I have also attached the Pdf marked with suggestions for the authors' kind perusal.

**Response: Thanks for the same; we have addressed all the markings.**
