# Supplementary material for: Early onset of aridity in the past millennium: Insights from vegetation dynamics and climate change in the alpine, cold-desert region of Trans Himalaya, India
Source: PLoS One. 2024 Jan 10;19(1):e0295785. doi: 10.1371/journal.pone.0295785 (PMC10781162; doi:10.1371/journal.pone.0295785)
Supplement: Attachment — Submitted filename: Response to Reviewers.doc [file pone.0295785.s010.doc]

**Response to Reviewers**

**Reviewer # 1**

At the outset, may we most humbly submit that if our responses ‘have appeared to be rather combative than professional’, then we do beg your pardon. Perhaps, in our attempt to profess our points of view, we might have been somewhat overzealous. Hope you will bear with us and excuse us for the same.

As suggested, we revisited and had a look at the earlier comments and we sincerely believe that we had addressed the queries to the best of our abilities. Nonetheless, we would surely like to further resolve the issues and address your main concerns related to:

*Early onset of aridity*: Please allow us to say that in our opinion we have the required palynological data, backed by mineral magnetic and geochemical proxies to propose an early onset of cold amd arid conditions in the region. At 950 yr BP we find:

a. A sharp decline in the AP/NAP ratio (from 82% to 65%).
b. Proliferation of arid-loving steppe elements, specially *Artemesia*, and a consequent decline in moist-loving ferns.
c. Low magnetic susceptibility, indicating less weathering as a result of prevailing aridity.
d. Increase in geochemical indices, implying cold-arid conditions.

We have discussed these findings comprehensively under Results and interpretations; sub-head Palynological analysis of the sub-surface sediments; Pollen zone-III; and also Discussion: 950 yr BP to the Present, 1^st^ paragraph.

Also, as suggested by you in your earlier comment that ‘as to why cannot it be interpreted as a period of aridity within MCA? Yes, your observation is fine and we have incorporated it in the text as well (please see Discussion, 1330 to 950 yr BP, first paragraph). But even then, aridity has crept in early in the region, during the MCA itself.

Besides, your earlier observation that – “Does this mean that the authors suggest beginning of LIA prior to termination of MCA? So taking your suggestion forward, we understand that the LIA had begun only after the termination of the MCA. Required modifications have been done in the text (Abstract and Discussion). Also we are further discussing the issue below:

*Duration of LIA and the termination of MCA*: We understand that there might have been some confusion regarding the duration of LIA vis-à-vis the termination of MCA, in the way we had presented these in our paper. We have now clearly stated that the LIA commences from 680 yr BP (AD 1270), after the termination of the MCA (please note that the commencement of the LIA at 680 yr BP (AD 1270) in the present region, is rather early if compared with the generally established global records (LIA: AD 1500 to 1850). Suitable modifications have been made in the text (please see Discussion, 950 yr BP to the Present, second paragraph; Conclusion).

We earnestly hope that we have now addressed the main issues and have been able to resolve the discrepancies. Looking forward to your benevolent support on our findings.
